# **Prevalence of mental disorders among young people living in urban slums of low- and middle- income countries: A systematic review and Meta-analysis**

**DOI:** 10.1007/s00127-026-03154-9

**Published:** 2026-06-26

**Authors:** Tolulope Bella-Awusah, Oluwabunmi Fola – Bolumole, Sagar Jilka, Ursula Read, Olayinka Omigbodun, Swaran Singh, Domenico Giacco

**Affiliations:** 1 Applied Health Division, Warwick Medical School, University of Warwick, Coventry, CV4 7AL UK; 2 Department of Psychiatry, University College Hospital Ibadan, Ibadan, Nigeria; 3 Centre for Child and Adolescent Mental Health & Department of Psychiatry, College of Medicine, University of Ibadan, Ibadan, Nigeria; 4 School of Health and Social Care, University of Essex, Essex, UK

**Keywords:** Young people, Mental Health, Urban Slums, Prevalence, Systematic Review, Low- and- middle income countries

## Abstract

**Purpose:**

Young people living in slums are at increased risk of poor mental health. We examined the prevalence and correlates of mental disorders among youth living in slums in low-and middle-income countries (LMICs).

**Methods:**

A search of seven databases (MEDLINE, Embase, PsycINFO, Scopus, Web of Science, CINAHL, and African Journals Online) was conducted from inception to September 2022 and updated to March 2026. Studies were screened by two independent reviewers who also extracted data and assessed the study quality and risk of bias using the Joanna Briggs Institute critical appraisal tool (JBI). Meta–analyses were carried out using the random effects model (REML) to estimate prevalence and 95% CI.

**Results:**

We identified 24 studies out of 3,946 citations, reporting data on depression, post- traumatic stress disorder (PTSD), anxiety, and alcohol use disorders (AUDs), involving 26,794 participants. The pooled prevalences were depression 31% (95% CI: 0.23–0.40; I^2^ 99·4%; *p* < 0·01), PTSD 24% (95% CI: 0.14–0.35; I^2^ 99·08%; *p* < 0·01), anxiety disorders 35% (95% CI: 0.20–0.53, I^2^ 98·91%; *p* < 0·01), AUDs 23% (95% CI:0.10–0.38, I^2^ 99·08%; *p* < 0·01). Food insecurity and violence exposure were the most reported risk factors, whereas adult support and neighbourhood connectedness were the most reported protective factors.

**Conclusion:**

Young people living in slums carry a disproportionate burden of mental disorders. There is an urgent need for contextually grounded policies and interventions to mitigate identified risk factors, strengthen protective factors, and break down barriers to treatment for those in need.

**Supplementary Information:**

The online version contains supplementary material available at https://doi.org/10.1007/s00127-026-03154-9.

## Introduction

Mental disorders are one of the leading causes of disability among young people globally, and nearly half of all lifetime mental disorders emerge by the age of 14 [1, 2]. Despite this, most affected youth particularly those who live in low- and middle-income countries (LMICs) often do not receive formal diagnoses or treatments [3, 4]. This treatment gap is often greatest among those already facing greater structural disadvantages such as those living in informal urban settlements known as slums [5, 6].

Globally, an estimated one billion people live in slum settlements, and it is projected that this figure will continue to rise as urbanisation accelerates across sub-Saharan Africa and South Asia [7–9]. Young people make up a large proportion of slum dwellers, yet they remain largely invisible in the mental health literature. Life in slums is often characterised by a concentration of chronic adversities which have well-established associations with poor mental health [10]. Extreme poverty, overcrowding, inadequate sanitation, food insecurity, exposure to community violence, disrupted schooling, and limited access to health services all co-occur in slum environments [8, 11, 12] where they interact synergistically, leading to compounded risks across biological, psychological, and social spheres of life [13, 14]. The resulting ecology of adversity in slums therefore appears distinct from non-slum urban or rural ecologies, and should not be treated as a subcategory of either [13].

A growing body of research has begun to examine mental health outcomes among youth living in slum settings (YLS), with reports of elevated rates of depression, anxiety, post-traumatic stress disorder (PTSD), and alcohol and substance use disorders [15–17]. However, the available evidence remains largely fragmentated making it difficult to draw reliable conclusions about the true prevalence of mental disorders in this population, and to identify risk and protective factors which could be targets of interventions. Therefore, this systematic review aims to address a critical gap in the global mental health literature by synthesising evidence on the prevalence and correlates of mental disorders among YLS.

## Methods

### Protocol and registration

This systematic review and meta-analysis was conducted according the PRISMA guidelines [18]. The review protocol was registered with PROSPERO (ID number CRD42022359756).

## Eligibility criteria

The inclusion criteria were as follows: studies reporting point prevalence or period prevalence of up to 12 months on disorders from F1 to F4 on the ICD-10 classification of mental disorders; studies that used validated diagnostic and/or self-report instruments; study population had to reside in an urban slum area as identified by the article authors; or the study provided sufficient information to determine whether households lacked one or more of the five indicators used by UN-HABITAT [19] to identify slum households. These five indicators are (1) insecure residential status, (2) poor structural quality of housing, (3) overcrowding, (4) inadequate water access, and (5) inadequate sanitation access. Eligible studies could be observational, cross-sectional, case-control, cohort studies, randomized controlled trials (RCT), and nonrandomized controlled trials (nRCT), if they reported the prevalence of the selected disorders in the selected population. Studies using secondary data or based in clinical settings of LMICs were excluded. LMICs were identified based on World Bank classification [20].

## Search strategy

The following seven databases were searched: MEDLINE, Embase, PsycINFO, SCOPUS, Web of Science, CINAHL, and African Journals Online. OpenGrey was listed as a planned grey literature source in the registered protocol but was not searchable due to discontinuation of the source. The original search was conducted on 1 September 2022. Search terms relating to youth, mental health, poor urban environments, and LMICs were developed in collaboration with an information specialist. The reference lists of included papers were manually searched for additional studies. There were no restrictions on search dates or language. During the peer review period, the search was updated to 12 March 2026 to ensure that the evidence base was up to date. The search strategy was expanded to include additional terms for ‘prevalence’ and ‘poor urban environments’ to improve comprehensiveness. (Supplement 1).

## Study selection

After removing duplicates, the titles and abstracts were screened by two independent reviewers (TB-A and OF-B). If the title and abstract did not provide sufficient information to determine eligibility, the article was retained for a full-text screening. The full texts of the articles that were found eligible or unclear at this stage were retrieved and screened again by two independent reviewers. Disagreements between reviewers were resolved through discussion or in cases of persistent disagreement, by a third adjudicating reviewer (DG).

## Data extraction and preparation

Two independent reviewers, TB-A and OF-B A used a standardised Excel spreadsheet to extract data from all included studies. The following data were extracted from each study: author and year, country, study design, sampling method, age range, mean age, sex ratio, mental disorder(s), assessment tools, diagnostic criteria, total sample size, risk factors, and protective factors.

### Risk of bias assessment

The risk of bias assessment of the included studies was performed by two independent reviewers, TB-A and OF-B, using the Joanna Briggs critical appraisal tool (JBI) for prevalence studies [21]. The checklist rates studies using nine criteria, such as appropriateness of the sampling frame, sampling method, sample size, rigour of data analysis, clear target population definition, appropriate measurement, valid/reliable tools, appropriate statistical analysis, and response rate. Each question is rated as *Yes*,* No*,* Unclear* or *Not Applicable*. Each Yes response was assigned a score of one point. Thus, the studies were scored on a scale of 0–9 points (Supplement 2).

### Data analysis

The extracted data were imported into Stata software version 18 (StataCorp LP, College Station, TX, USA) for meta-analysis. Meta-analysis results were expressed as prevalence estimates of mental disorders calculated with 95% CIs in the pooled data. The Random effects model was used, assuming heterogeneity among the studies (using the REML model). This statistical model assumes that the samples of the included studies were drawn from different populations. Heterogeneity was assessed using the I^2^ statistic. The I^2^ value provides a measure of the variation explained by the differences between the included studies rather than by chance [22]. Possible sources of heterogeneity between studies were investigated, where reported data allowed subgroup analysis using the Meta suite in Stata 18.0. These included age, region, sampling method, risk of bias, study instruments, and mode of administration of the assessment tool. A narrative synthesis was also conducted on the individual-level correlates of the mental disorders identified in the review.

## Results

### Search results

**Fig. 1:**
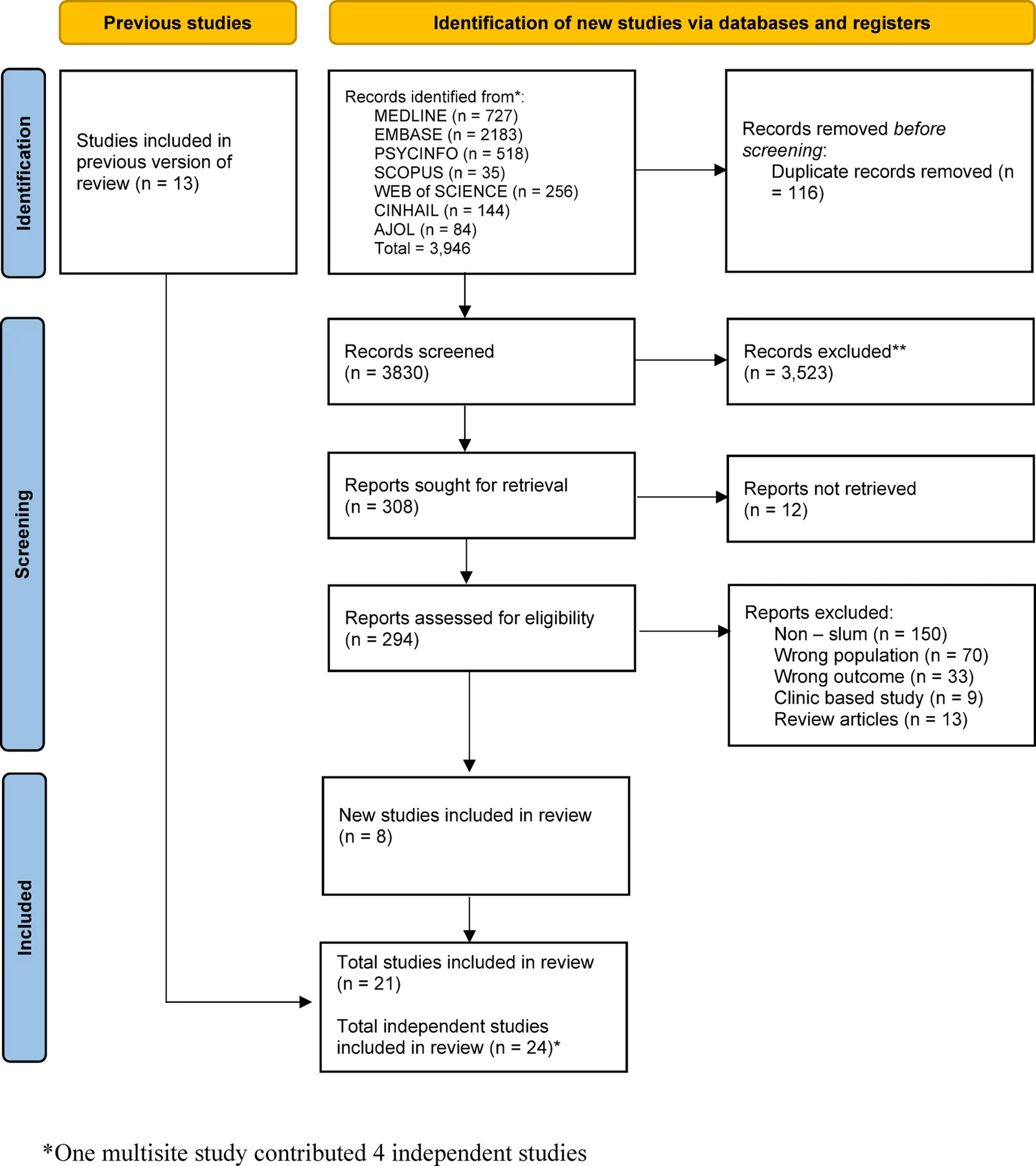
***PRISMA 2020 flow diagram***

**Fig. 2:**
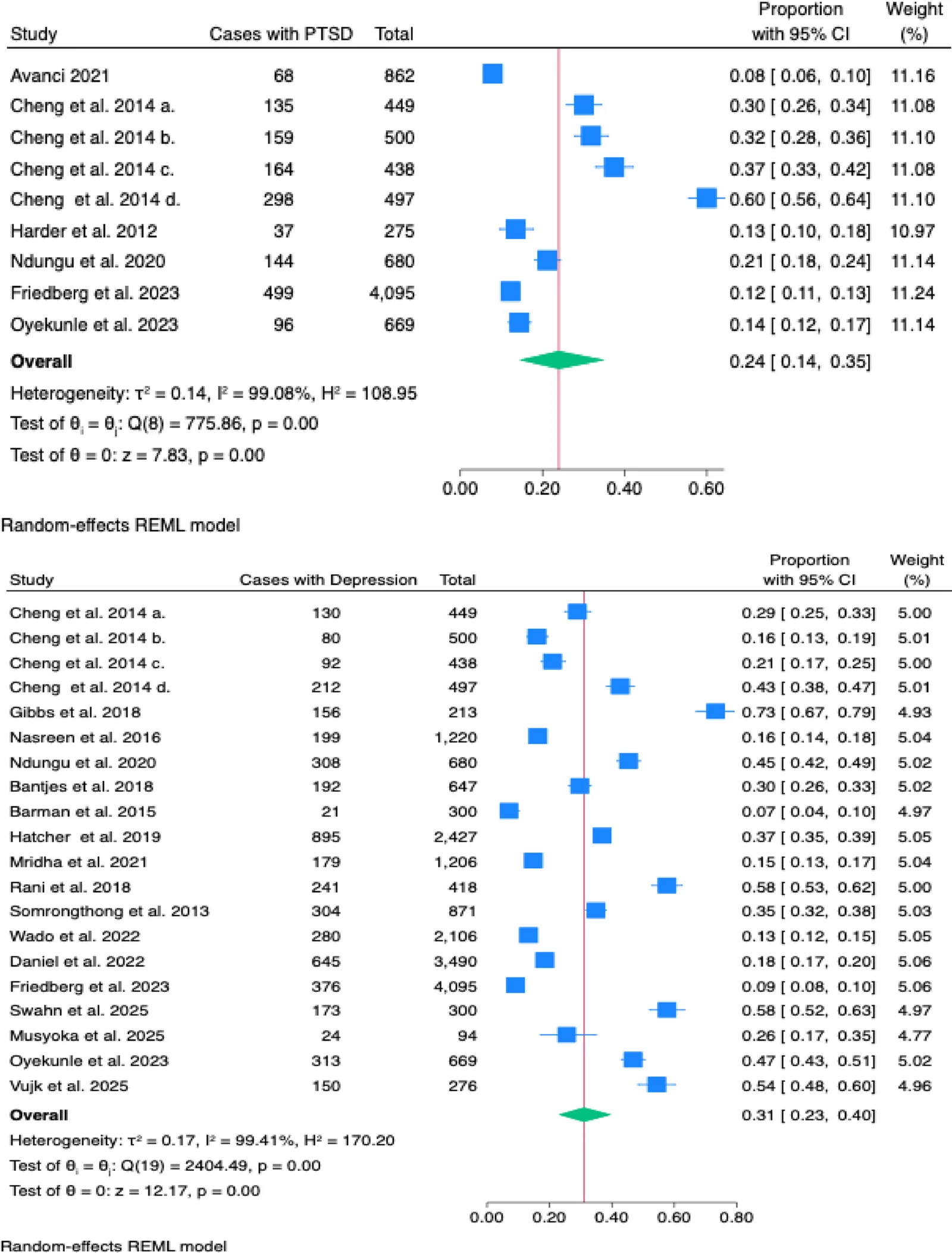
Forest plots of pooled prevalence for Depression and PTSD

Figure. 1 A search of the seven databases retrieved 3,946 citations for review. These were imported into the Endnote 25 reference manager, and 116 duplicates were removed, leaving 3,830 articles for screening. These were then imported into the Rayyan Artificial Intelligence software [23] for title and abstract screening. A total of 308 articles were included for full-text screening, and 21 articles were finally included in the review (Fig. 2). One article reported a multisite study with four independent samples, each treated as a separate study in the review resulting in 24 independent studies.

### Characteristics of the included studies

Twenty-one articles (comprising 24 independent studies) were included in the review [15, 24–43]. All the articles were published between 2012 and 2026. The studies were from three regions: thirteen from Africa (Nigeria (*n* = 1), South Africa (*n* = 7), Kenya (*n* = 4), Uganda (*n* = 1), nine from Asia (India (*n* = 5), China (*n* = 1), Bangladesh (*n* = 2), Thailand (*n* = 1), and two from South America (Brazil and Paraguay).

A total, 26,794 participants were included in this review. Of these, 11, 748 were males and 14,769 were females and 277 were from one study which did not report the sex breakdown. The studies reporting sex breakdown resulted in a female proportion of 0·55. All studies were cross-sectional in design, and 13 used non-probability sampling methods (Table 1). The ages of the participants ranged from 12 to 30 years, but only six studies specified the mean age and SD of the sample population. Five studies had 100% female participants, and three studies had 100% male participants.

Nine studies examined PTSD using data from 8,465 participants [15, 24, 27, 29, 35, 36]. Twenty studies examined depression using data from 20, 896 participants [25–30, 33–43]. Six studies examined anxiety disorders with data from 5,483 participants [26, 29, 33, 37, 39, 41], and five studies examined alcohol use disorders with data from 6,323 participants [30–32, 35, 36]. One study examined tobacco and cannabis use disorders [32]. Only one study used a clinician’s rating scale in addition to a screening scale [15]. All other studies used self-rated or interviewer-rated, validated screening scales.

For depression, 9 studies [25–27, 30, 36, 38] used the Centre for Epidemiological Studies Depression scale CES-D 10 item and 20 item versions [44]. One study [34] used the Beck Depression Inventory (BDI) [45], one study used the Beck child depression inventory (CDI) [29], five studies [28, 33, 40, 41, 43] used the Patient Health Questionnaire (PHQ- 9) [46], one study [37] used the Mental Health Inventory (MHI) [47], one study [26] used the Kutcher Adolescent Depression Scale (KADS) [48], and one study [39] used the Depression and Anxiety Stress Scale [49].

For PTSD, four studies [27] used the PTSD checklist civilian version – the 6-item short form [50], and two studies [15, 24] used the University of California at Los Angeles (UCLA) PTSD reaction index [51]. One study [15] used both the UCLA PTSD reaction index and the clinician-administered PTSD scale [52]. Two studies [35, 36] used the Harvard Trauma Checklist [53] and one study [29] used the Child PTSD symptom scale [54].

For anxiety, one study [37] used the Mental Health Inventory (MHI), one study [39] used the Depression and Anxiety Stress Scale [49], three studies [26, 33, 41] used the Generalised Anxiety Disorder Scale (GAD-7) [55], and one study [29] used the Beck’s Anxiety Inventory [56]. For Alcohol Use Disorders (AUD), four studies [30, 31, 35, 36] used the Alcohol Use Disorders Identification Test (AUDIT) [57] and one study [32] used the WHO Alcohol, Smoking and Substance Involvement Screening Test (ASSIST) [58].

A total of 13 studies [15, 27–29, 31, 33, 36, 38, 41, 43] mentioned cultural adaptations of the screening instruments used, while 15 studies [15, 27, 28, 31, 33, 35–38, 40–42] mentioned administration in the local language in addition to or alternatively to the English language. Studies with JBI scores of less than 6 were rated as moderate to high levels of bias, and nine out of the 24 studies met this criterion. All studies were included in the meta-analysis regardless of quality.

### Prevalence of mental disorders

**Fig. 3:**
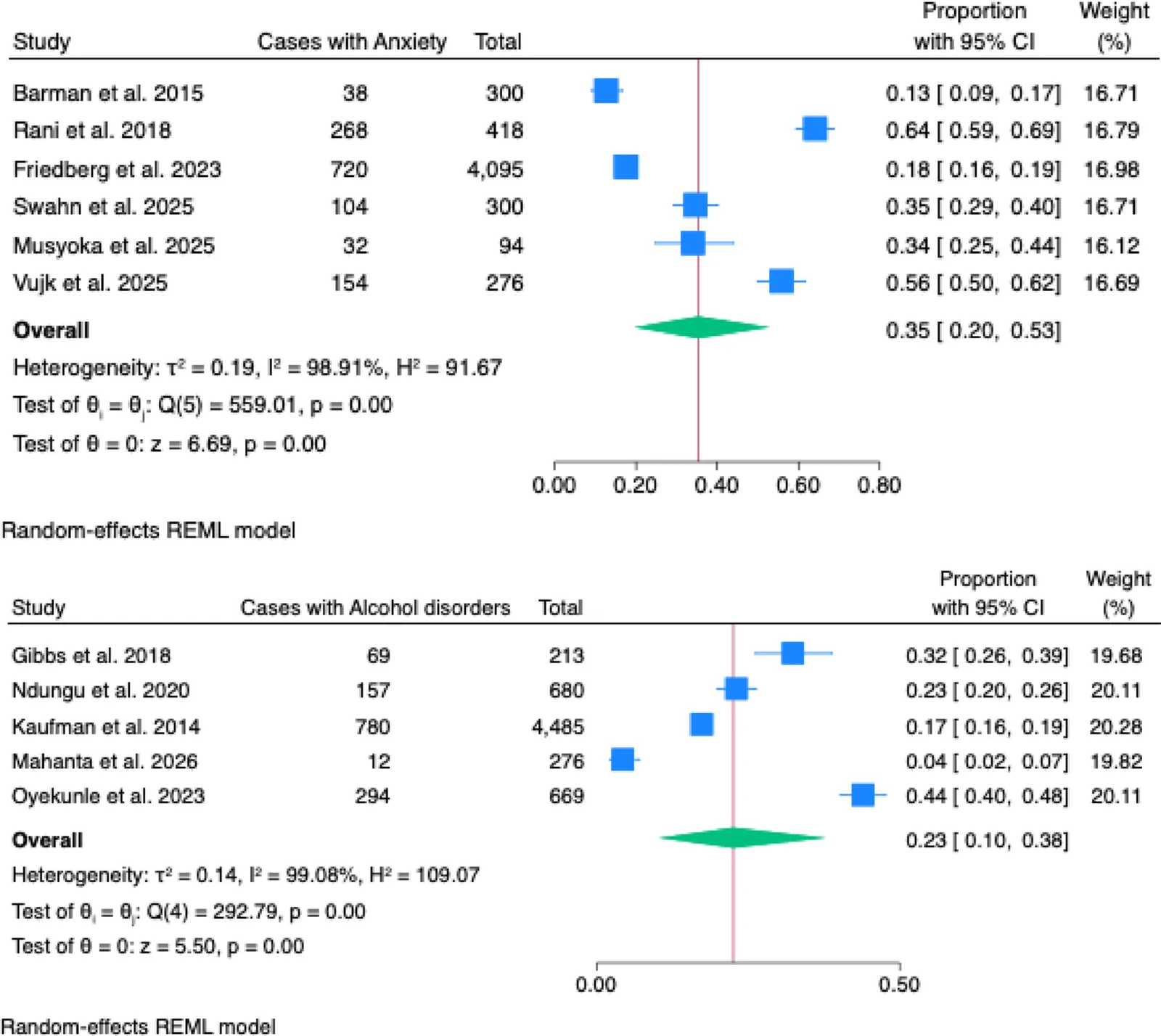
Forest plots of pooled prevalence for Anxiety and Alcohol use disorders

The pooled prevalence for depression was 31% (95% CI: 0.23–0.40, *N* = 20,896), with a range of 7·0–73.0%, and heterogeneity I^2^ of 99·4% (*p* < 0·01), (Fig. 2).The pooled prevalence for PTSD was 24% (95% CI: 0.14–0.35, *N* = 8,465), with a range of 8.0–60.0%, and heterogeneity I^2^ of 99·08% (*p* < 0·01) (Fig. 2). The pooled prevalence of anxiety disorders was 35% (95% CI: 0.20–0.53, *N* = 5,483), with a range of 13.0–64.0%, and heterogeneity I^2^ of 98·91% (*p* < 0·01) (Fig. 3). The pooled prevalence for alcohol use disorders was 23% (95% CI:0.10–0.38, *N* = 6,323), with a range of 4.0–44.0%, and heterogeneity I^2^ of 99·08% (*p* < 0·01) (Fig. 3). One study [32] reported prevalence of tobacco and cannabis disorders as 13% and 0.7% respectively.

**Table 1:** Characteristics of included studies

S/*N*	Author	Year	Region	Country	Study design	Sampling method	Age range	Gender Ratio (female)	Sample size	Mental disorders	Assessment Tool	Mode of administration
1.	Avanci 2021	2021	South America	Brazil	Cross sectional	Multistage cluster sampling	13 to 19	65	862	PTSD	UCLA PTSD reaction Index	Self
2.	Cheng et al. 2014 a.	2014	Africa	Nigeria	Cross sectional	Respondent driven sampling	15 to 19	51	449	PTSD Depression	CESD − 10; (cut off 11) PTSD checklist civilian version	Self
3	Cheng et al. 2014 b.	2014	Asia	India	Cross sectional	Respondent driven sampling	15 to19	50	500	PTSD Depression	CESD − 10; PTSD checklist civilian version	Self
4	Cheng et al. 2014 c.	2014	Asia	China	Cross sectional	Respondent driven sampling	15 to19	49.3	438	PTSD Depression	CESD − 10; PTSD checklist civilian version	Self
5	Cheng et al. 2014 d.	2014	Africa	South Africa	Cross sectional	Respondent driven sampling	15 to 19	45.2	497	PTSD Depression	CESD − 10; PTSD checklist civilian version	Self
6	Gibbs et al. 2018	2018	Africa	South Africa	Cross sectional	Snowball sampling	18 to 30	49.8	213	Depression AUD	CES D −20 (cut off 16) AUDIT	Self
7	Harder et al. 2012	2012	Africa	Kenya	Cross sectional	Stratified random sampling	12 to 18	53.5	275	PTSD AUD	UCLA PTSD reaction index; Clinician PTSD scale; AUDIT	Clinician rated (PTSD), self
8	Nasreen et al. 2016	2016	Asia	Bangladesh	Cross sectional	Multiple random sampling	13 to 19	50	1220	Depression	Beck’s depression Inventory	Interviewer
9	Ndungu et al. 2020	2020	Africa	South Africa	Cross sectional	Purposive and snowball sampling	18 to 30	100	680	PTSD, Depression AUD	AUDIT (cut off 8); CESD: Harvard Trauma Questionnaire	Interviewer (DEP), self
10	Bantjes et al. 2018	2018	Africa	South Africa	Cross sectional	Systematic random sampling	18 to 29	0	647	Depression	CES-D 20 (cut off 16)	Interviewer
11	Barman et al. 2015	2015	Asia	India	Cross sectional	Systematic random sampling	15 to 19	100	300	Depression Anxiety	KUTCHER Depression scale; GAD − 7	Interviewer (DEP), self
12	Hatcher et al. 2019	2019	Africa	South Africa	Cross sectional	Cluster and convenience sampling	18 to 30	0	2427	Depression	CES-D −20 (cut off 21)	Interviewer
13	Mridha et al. 2021	2021	Asia	Bangladesh	Cross sectional	Cluster random sampling	10 to 19	50.6	1206	Depression	PHQ 9	Interviewer
14	Rani et al. 2018	2018	Asia	India	Cross sectional	Stratified two stage random sampling	13 to 19	100	418	Depression Anxiety	Mental Health Inventory MHI	Interviewer
15	Somrongthong et al. 2013	2013	Asia	Thailand	Cross sectional	Systematic random sampling	12 to 22	57.2	871	Depression	CES- D −20 (cut off 22)	Self
16	Wado et al. 2022	2022	Africa	Kenya	Cross sectional	Random sampling	12 to 19	100	2106		PHQ − 9	Self
17	Daniel et al. 2022	2022	Asia	India	Cross sectional	Census based purposive sampling	15 to 19	45.2	3490	Depression	PHQ − 9	Interviewer
18	Friedberg et al. 2023	2023	Africa	Kenya	Cross sectional	Cluster randomised trial	10 to 14	79.8	4095	PTSD Depression Anxiety	CPSS; CDI-2; BAI	Self
19	Kaufman et al. 2014	2014	Africa	South Africa	Cross sectional	Purposive cluster sampling	12 to 20	55.6	4484	AUD	AUDIT	Self
20	Swahn et al. 2025	2025	Africa	Uganda	Cross sectional	Convenience sampling	18 to 24	100	300	Depression Anxiety	PHQ- 9; GAD-7	Interviewer
21	Mahanta et al. 2026	2026	Asia	India	Cross sectional	Random sampling	12 to 19	49.2	276	AUD Tobacco use disorder Cannabis use disorder	WHO ASSIST	Self
22	Musyoka et al. 2025	2025	Africa	Kenya	Cross sectional	Snowball sampling	15 to 24	45.7	94	Depression Anxiety	PHQ − 9; GAD − 7	Interviewer
23	Oyekunle et al. 2023	2023	Africa	South Africa	Cross sectional	Snowball sampling	18 to 30	0	669	PTSD, Depression AUD	CES-D 20; Harvard Trauma Questionnaire; AUDIT	Interviewer
24	Vujk et al. 2025	2025	South America	Paraguay	Cross sectional	Purposive sampling	12 to 18	unknown	276	Depression Anxiety	DASS- 21	Self

### Sub-group analysis

**Fig. 4:**
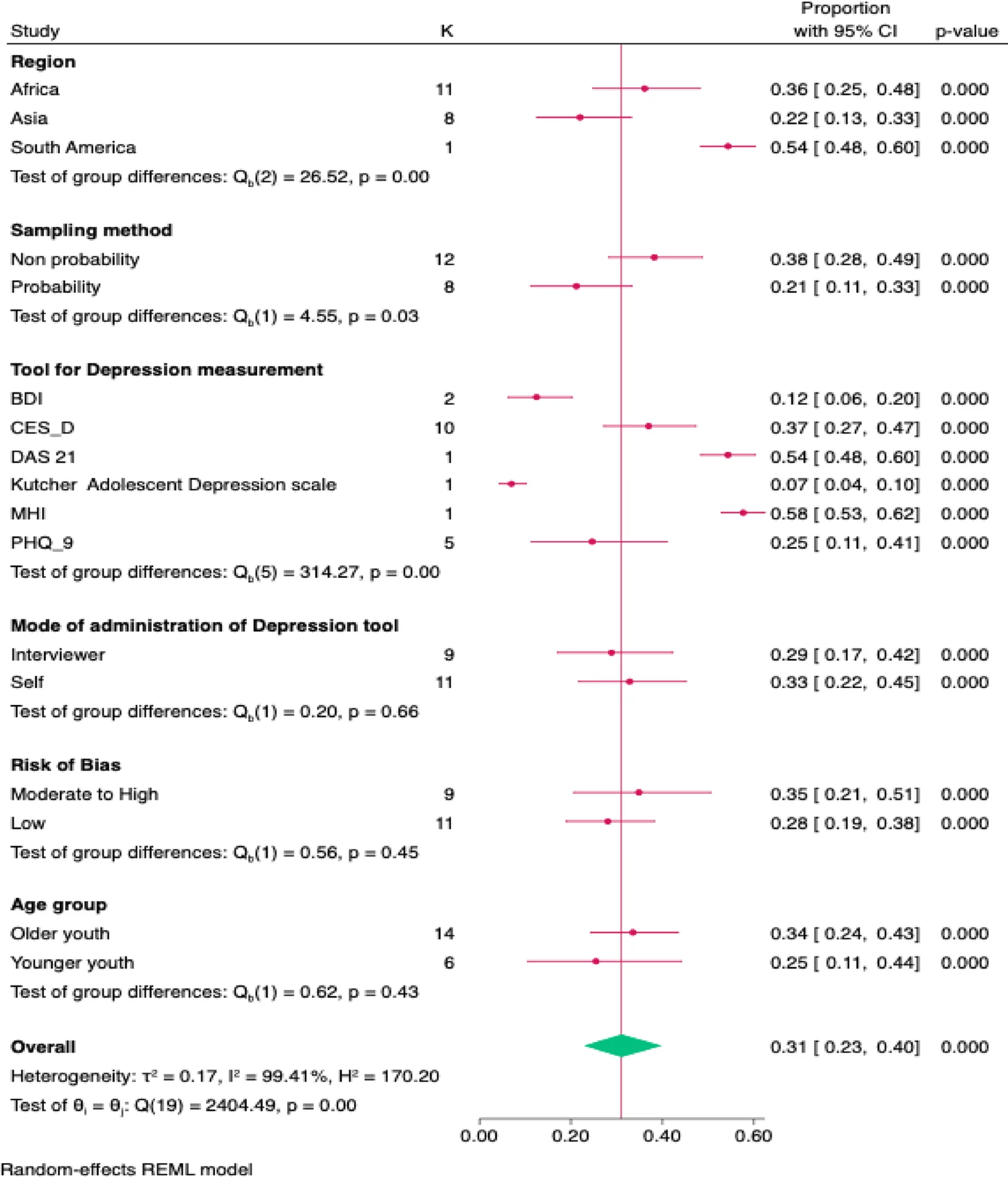

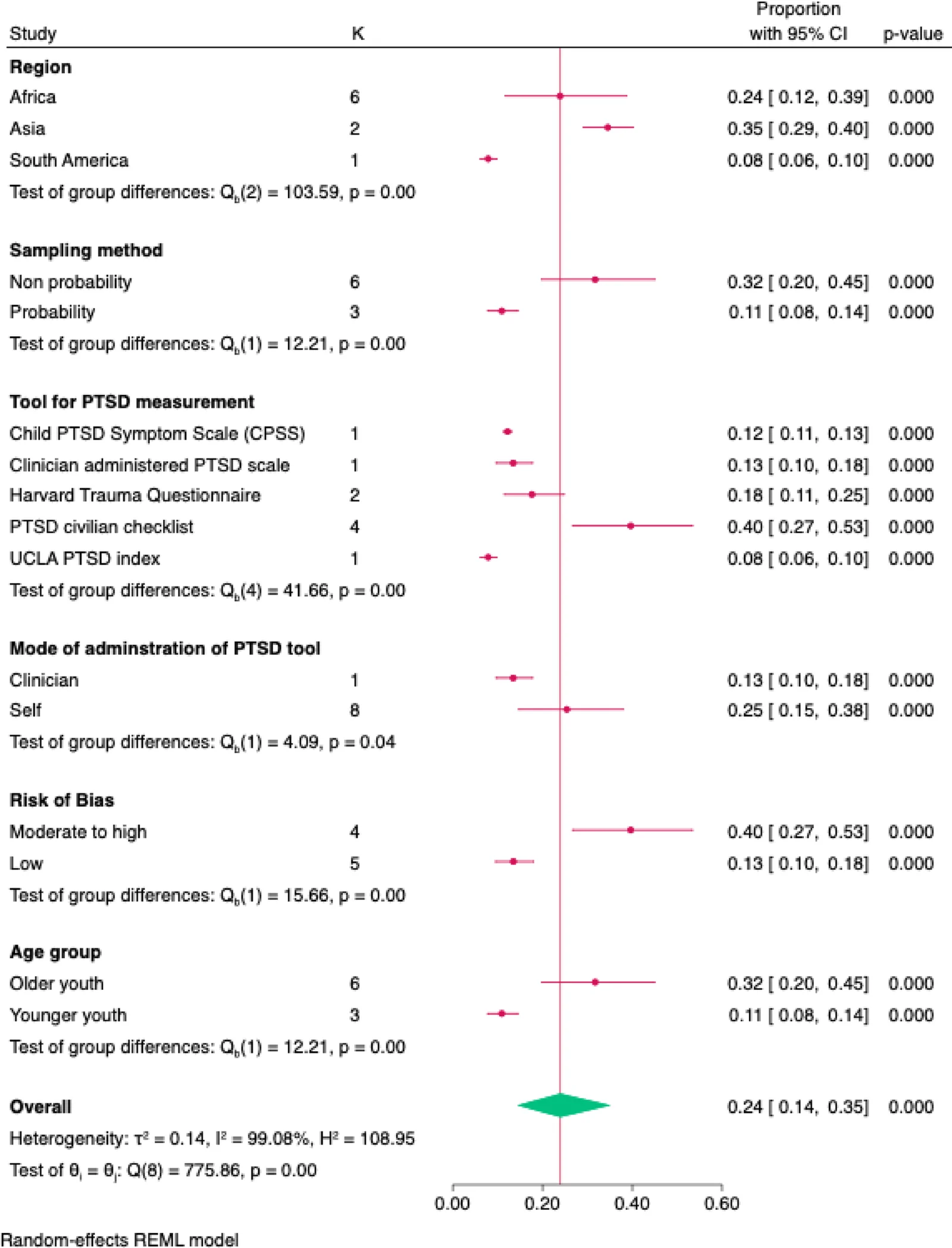
Forest plots showing subgroup analysis for Depression and PTSD

For depression, prevalence differed significantly by region (*p* < 0.001), sampling method (*p* = 0.03), and measurement tool (*p* < 0.001), but not by mode of tool administration, risk of bias, or age group. Regionally, South America had the highest prevalence (54%, 95% CI: 0.48 − 0.60), followed by Africa (36%, 95% CI: 0.25–0.48) and Asia (22%, 95% CI: 0.13–0.33). Non-probability sampling yielded higher estimates than probability sampling (38%, 95% CI: 0.28–0.49 vs. 21%, 95% CI: 0.11–0.33). Estimates varied widely by tool, ranging from 7%, 95% CI: 0.04–0.10 (Kutcher Adolescent Depression Scale) to 58%, 95% CI: 0.53–0.62 (Mental Health Inventory). For PTSD, all subgroup variables were significant moderators, including region, sampling method, measurement tool, mode of administration, risk of bias, and age group (all *p* ≤ 0.04). Asia had the highest regional prevalence (35%, 95% CI: 0.29 − 0.40), followed by Africa (24%, 95% CI: 0.12–0.39) and South America (8%, 95% CI: 0.06 − 0.10). Non-probability sampling also yielded higher estimates (32%, 95% CI: 0.20–0.45 vs. 11%, 95% CI: 0.08–0.14). Self-administered tools reported higher prevalence than clinician-administered tools (25%, 95% CI: 0.15–0.38 vs. 13%, 95% CI: 0.10–0.18), as did studies with moderate-to-high vs. low risk of bias (40%, 95% CI: 0.27–0.53 vs. 13%, 95% CI: 0.10–0.18). Older youth had higher PTSD prevalence than younger youth (32%, 95% CI: 0.20 − 0.45 vs. 11%, 95% CI: 0.08–0.14). See Fig. 4.

### Individual level correlates of mental disorders

Individual correlates of mental disorders were explored and extracted from 21 studies. These were calculated by the study authors using multivariate regression models at a p value of < 0·05.

### Depression

The risk factors identified for depression in both genders included food insecurity, poor relationships with parents, older age (> 15 years), female gender, high slum adversity score, and positive Covid 19 infection [28, 34, 59]. Stealing food, involvement in criminal activities, a family history of depression, being fired from work, dissatisfaction with income, financially supporting two or more dependents, problematic alcohol use, and childhood physical, psychological, and sexual abuse were also identified as risk factors for depression in males [25, 36, 42]. For females, physical, psychological, or sexual abuse, poor maternal connection, a history of adverse events in the family, perceiving the neighborhood as unsafe, being unmarried, being a homemaker, having a reproductive illness, being a victim of attempted rape or victim of ‘eve teasing’ (an Indian term for sexual harassment in a public place) all increased risk [29, 34, 40]. The protective factors for depression in males was a caring adult male in the home. A caring adult was defined as an adult who had high expectations of the young person such as following rules and being successful in their activities. These adults also listened to and helped youth as needed. Caring adult males and females in the home was protective for females [27]. Feeling connected to the neighborhood (defined as knowing most people and feeling part of the neighborhood) was protective for both genders [27]. Higher education and positive peer support (a friend who could be trusted and provided practical assistance, with unconditional acceptance) was protective for both genders [25, 28]. More positive feelings about work and less control of partners was also protective for males, whereas less stress at work was protective for females [27, 59].

### PTSD

The risk factors for PTSD across both sexes were older age, impaired social functioning, severe maternal physical violence, psychological violence by a significant other, witnessing one to two or more traumatic events, and being a victim of rape [24, 29],. Food insecurity and problematic alcohol use were also risk factors for males [36]. A protective factor for both sexes was feeling connected to their neighbourhood and completing secondary school education. Having a caring male or female adult in the home was protective only in males [27, 36].

### Anxiety Disorders

The risk factors for anxiety disorders in females included a history of rape, lower levels of education, lower social class, and food insecurity [26, 29, 37]. Protective factors were not reported in the included studies.

### Alcohol use disorders (AUDs)

Risk factors for alcohol use disorders included older age, male sex, drug use in the past year, non-partner sexual violence in the past year, transactional sex with a partner, depressive symptoms, being from a joint family, no parental education, being sexually active, two or more sexual partners in the last 12 months, and daily social media use [31, 32, 35]. Protective factors were not reported in the included studies.

### Publication Bias

Publication bias assessment was limited to depression, as it was the only disorder with a sufficient number of studies (*n* > 10) to allow for reliable testing. A funnel plot showed an asymmetric distribution, suggesting the presence of publication bias (Supplement 3). Egger’s test [60] did not reach statistical significance (β₁ = 7.77, 95% CI: −1.00 to 16.54, *p* = 0.08). However, given the borderline p-value and visual asymmetry of the funnel plot, publication bias cannot be fully excluded.

## Discussion

This review found data on four disorders: depression, PTSD, anxiety, and substance use disorder. Depression with a pooled prevalence of 31% with was comparable to the 0–28% among youth in LMICs [61], and the 34% among youth globally. Notably, LMIC and global figures include youth living in very difficult circumstances, such as those living with HIV, in areas of prolonged conflict, and orphaned youth [62]. While the rates of depression in this review were comparable with LMIC and global estimates, depression often occurs comorbidly with PTSD, Anxiety, and AUDs [63, 64] which were all elevated in this review. However, data on comorbid disorders was not available for extraction from the included studies.

PTSD with a pooled prevalence of 24% was approximately 1.5x higher than the 15.9% reported among trauma-exposed youth globally [65]. This pooled prevalence was also much higher than the 6.2–15.3% reported among trauma exposed youth in ten LMICs [66]. Older age was a risk factor for PTSD in this review, although a previous meta-analysis did not find age-related PTSD associations [64]. This association may reflect the increased vulnerability to mental health problems during puberty [2, 67], which when coupled with growing social responsibilities, academic, and economic pressures, may intensify stress responses increasing YLS vulnerability to PTSD [68]. Similar to previous studies, other risk factors for PTSD identified in this review were violence from significant others, and multiple traumatic exposures [61, 65]. These may increase PTSD risk by overwhelming YLS coping resources and weakening their sense of safety and trust [69, 70].

Anxiety with a pooled prevalence of 35% was higher than the 8–27% prevalence reported in other LMIC countries [61]. Social and economic factors common in slums such as chronic uncertainty, violence, and economic precarity [29, 37] are likely to increase anxiety risk among YLS beyond estimates reported in broader LMIC surveys, which typically combine urban and rural youth which may mask the concentrated adversity typically found in slum settlements. The pooled prevalence of AUD (23%) was also three times higher than the 7.7% reported in a previous sub-Saharan Africa meta-analysis [71]. Structural and economic factors specific to slum communities such as high youth unemployment, limited structured recreational activities, and the concentration of informal alcohol shops may create permissive environments for harmful use [72, 73]. Social norms around alcohol may also differ within slum communities, where drinking may function as a coping mechanism for chronic adversity and social stress in ways that sustain higher rates of its disordered use [74].

The most frequent risk factors in this review were food insecurity and violence exposure.

Food insecurity is often used as a proxy for poverty [75], and an increasing body of research has explored the association between food insecurity and youth mental health among youth in LMICs [40, 76–78]. However, interventions addressing food insecurity have largely focused on nutritional and cognitive outcomes during early life [79]. Our findings suggest the need to consider mental health as an important outcome for interventions targeting food insecurity among youth in LMICs [80].

### Strengths and limitations

A key strength of this review was its systematic synthesis of data across diverse geographical, cultural, and socioeconomic contexts, highlighting global patterns and context-specific variations. To the best of our knowledge, this is the first review of its kind. However, it also has several limitations. Although no restrictions were applied to publication dates or language during the search, all studies that met the inclusion criteria were published in English. Thus, the findings from non-English-speaking slum settings are likely underrepresented. Representation across the globe was also not as diverse as expected with only two studies from South America and seven from South Africa.

There were wide variations in the study assessment tools which were all developed in high-income countries, though validated and adapted for LMIC settings. All studies except one, exclusively utilised screening instruments which may under or overestimate the true burden of mental disorders in LMIC slum settings due to differences in cultural idioms of distress, and stigma [81, 82]. Screening instruments may however detect subclinical symptoms which may also cause substantial distress and functional impairment. Capturing these symptoms is therefore valuable as they may represent important targets for early intervention. Overall, considering these limitations and the substantial heterogeneity among studies, the review findings should be interpreted with caution.

### Implications for policy and research

The high burden of mental disorders and AUDs among YLS makes a strong case for including mental health in urban development and slum upgrading policies. Social protection policies addressing poverty, food insecurity, and violence should recognise their mental health implications. National mental health policies should address the heightened vulnerability of YLS by ensuring their visibility in resource allocation and service planning. Mental health services for YLS should be community-based, culturally acceptable, and delivered by trusted actors. Interventions should focus on reducing risk exposure while building protective resources. Future research should prioritise longitudinal designs for better understanding of the associations between slum-related adversities and mental health outcomes. The use of standardised measurement approaches, as well as validated diagnostic tools, could help to improve comparability across studies. Engaging young people as active participants in research design and implementation is also a priority.

## Conclusion

Young people living in slum settlements face a disproportionate burden of mental health disorders. There is an urgent need for culturally responsive, contextually grounded policies and interventions which address identified risk factors such as violence exposure, and food insecurity, while strengthening the protective factors that buffer against adversity. Translating this evidence into action will require coordinated investments across health, education, social protection, and urban planning sectors.

## Supplementary Information

Below is the link to the electronic supplementary material.

Supplementary Material 1

## Data Availability

Data is provided within the manuscript and supplementary information files at the University of Warwick institutional repository: http://go.warwick.ac.uk/wrap.
