# Supplementary material for: Prevalence of mental disorders among young people living in urban slums of low- and middle- income countries: A systematic review and Meta-analysis
Source: Soc Psychiatry Psychiatr Epidemiol. 2026 Jun 26;61(9):1687–701. doi: 10.1007/s00127-026-03154-9 (PMC13578088; doi:10.1007/s00127-026-03154-9)
Supplement: Supplementary file 1 [file 127_2026_3154_MOESM1_ESM.docx]

**Manuscript title: Prevalence of Mental Disorders among Young People Living in Urban Slums of Low- and Middle- Income Countries: A Systematic Review and Meta-Analysis Submission ID: 164bb7e9-65c5-4458-aca0-3f9542a15ed6**

Tolulope Bella-Awusah ^1,3^ Oluwabunmi Fola – Bolumole ^3^ Sagar Jilka ^1^ Ursula Read ^4^ Olayinka Omigbodun ^3^ Swaran Singh ^1^ Domenico Giacco ^1^

^1^Applied Health Division, Warwick Medical School, University of Warwick, Coventry, UK

^2^ Department of Psychiatry, University College Hospital Ibadan, Ibadan, Nigeria

^3^Centre for Child and Adolescent Mental Health & Department of Psychiatry, College of Medicine, University of Ibadan, Ibadan, Nigeria

^4^ School of Health and Social Care, University of Essex, Essex UK

Correspondence to: Dr. Tolulope Bella-Awusah, Applied Health Division, Warwick Medical School, University of Warwick, Coventry, CV4 7AL, UK

[bellatolu@gmail.com](mailto:bellatolu@gmail.com); tolulope.bella-awusah@warwick.ac.uk

**SUPPLEMENT 1**

Search strategy for Databases

| DATABASE | SEARCH TERMS |
| --- | --- |
| MEDLINE | 1 exp Epidemiology/ or prevalence*.mp.  2 Incidence.mp.  3 Proportion*.ti,ab.  4 Occurrence.ti,ab.  5 Frequency*.ti,ab.  6 Rate*.ti,ab.  7 1 or 2 or 3 or 4 or 5 or 6  8 exp Mental Disorders/ or mental illness.mp.  9 exp Poverty Areas/ or exp Poverty/ or exp Developing Countries/ or slum  10 exp Urban Environments/ or informal settlement*.mp.  11 Inner city*.mp.  12 Favela.mp.  13 exp Marginalization/ or marginalisation.mp  14 shanty town.mp.  15 squatter settlement*.mp.  16 9 or 10 or 11 or 12 or 13 or 14 or 15  17 7 and 8 and 16  18 limit 7 to ("adolescent (13 to 18 years)" or "young adult (19 to 24 years)  19 "Low and middle income countries" OR "developing countries" OR BRICS OR BRIC OR Africa OR "north Africa" OR "northern Africa" OR "Africa South of the Sahara" OR "Central Africa" OR "east Africa" OR "Eastern Africa" OR "Southern Africa" OR "Western Asia" OR "central Asia" OR "southeastern Asia" OR "Caribbean" OR "West Indies" OR "South America" OR "Latin America" OR "Central America" OR Afghanistan OR Albania OR Algeria OR "American Samoa" OR Angola OR "Antigua and Barbuda" OR Argentina OR Armenia OR Azerbaijan OR Bahrain OR Bangladesh OR Barbados OR Benin OR Belarus OR Belize OR Bhutan OR Bolivia OR Bosnia-Herzegovina OR Botswana OR Brazil OR Bulgaria OR "Burkina Faso" OR Burundi OR Cambodia OR Cameroon OR "Cape Verde" OR "Central African Republic" OR Chad OR Chile OR China OR Colombia OR Comoros OR Congo OR "Costa Rica" OR "Cote d'Ivoire" OR Croatia OR Cuba OR Cyprus OR Czechoslovakia OR "Czech Republic" OR Slovakia OR Djibouti OR "Democratic Republic of the Congo" OR Dominica OR "Dominican Republic" OR "East Timor" OR Ecuador OR Egypt OR "El Salvador" OR Eritrea OR Estonia OR Ethiopia OR Fiji OR Gabon OR Gambia OR "Georgia" OR Ghana OR Grenada OR Guatemala OR Guinea OR "Guinea-Bissau" OR Guam OR Guyana OR Haiti OR Honduras OR Hungary OR India OR Indonesia OR Iran OR Iraq OR Jamaica OR Jordan OR Kazakhstan OR Kenya OR Korea OR Kosovo OR Kyrgyzstan OR Laos OR Latvia OR Lebanon OR Lesotho OR Liberia OR Libya OR Lithuania OR Macedonia OR Madagascar OR Malaysia OR Malawi OR Mali OR Malta OR Mauritania OR Mauritius OR Mexico OR Micronesia OR "Middle East" OR Moldova OR Mongolia OR Montenegro OR Morocco OR Mozambique OR Myanmar OR Namibia OR Nepal OR "Netherlands Antilles" OR "New Caledonia" OR Nicaragua OR Niger OR Nigeria OR Oman OR Pakistan OR Palau OR Panama OR "Papua New Guinea" OR Paraguay OR Peru OR Philippines OR Poland OR Portugal OR "Puerto Rico" OR Romania OR Russia OR Rwanda OR "Saint Kitts and Nevis" OR "Saint Lucia" OR "Saint Vincent and the Grenadines" OR Samoa OR "Saudi Arabia" OR Senegal OR Serbia OR Montenegro OR Seychelles OR "Sierra Leone" OR Slovenia OR "Sri Lanka" OR Somalia OR "South Africa" OR Sudan OR Suriname OR Swaziland OR Syria OR Tajikistan OR Tanzania OR Thailand OR Togo OR Tonga OR "Trinidad and Tobago" OR Tunisia OR Turkey OR Turkmenistan OR Uganda OR Ukraine OR Uruguay OR USSR OR Uzbekistan OR Vanuatu OR Venezuela OR Vietnam OR Yemen OR Yugoslavia OR Zambia OR Zimbabwe  20 18 and 19  Hits - 727 |
| **Embase** | 1 exp Epidemiology/ or prevalence*.mp. 6068210  2 Incidence.mp. 1913077  3 proportion*.ti,ab. 1348402  4 Rate*.ti,ab. 5887217  5 Occurrence.ti,ab. 733508  6 Frequency*.ti,ab. 1554064  7 1 or 2 or 3 or 4 or 5 or 6 12887396  8 exp Mental Disorders/ or mental illness.mp. 3536834  9 exp Poverty Areas/ or exp Poverty/ or exp Developing Countries/ or slum*.mp. 175303  10 exp Urban Environments/ or informal settlement*.mp. 1346  11 Inner city*.mp. 12830  12 Favela.mp. 121  13 exp Marginalization/ or marginalisation.mp. 1106  14 Shanty town.mp. 84  15 Squatter settlement*.mp. 190  16 9 or 10 or 11 or 12 or 13 or 14 or 15 189691  17 7 and 8 and 16 9507  18 limit 17 to (adolescent <13 to 17 years> or adult <18 to 64 years>) 5357  19 ("Low and middle income countries" or "Low income countries" or "developing countries" or BRICS or BRIC or Africa or "north africa" or "northern Africa" or "Africa South of the Sahara" or "Central Africa" or "east Africa" or "Eastern Africa" or "Southern Africa" or "Western Asia" or "central Asia" or "southeastern Asia" or "Caribbean" or "West Indies" or "South America" or "Latin America" or "Central America" or Afghanistan or Albania or Algeria or "American Samoa" or Angola or "Antigua and Barbuda" or Argentina or Armenia or Azerbaijan or Bahrain or Bangladesh or Barbados or Benin or Belarus or Belize or Bhutan or Bolivia or Bosnia-Herzegovina or Botswana or Brazil or Bulgaria or "Burkina Faso" or Burundi or Cambodia or Cameroon or "Cape Verde" or "Central African Republic" or Chad or Chile or China or Colombia or Comoros or Congo or "Costa Rica" or "Cote d'Ivoire" or Croatia or Cuba or Cyprus or Czechoslovakia or "Czech Republic" or Slovakia or Djibouti or "Democratic Republic of the Congo" or Dominica or "Dominican Republic" or "East Timor" or Ecuador or Egypt or "El Salvador" or Eritrea or Estonia or Ethiopia or Fiji or Gabon or Gambia or "Georgia" or Ghana or Grenada or Guatemala or Guinea or "Guinea-Bissau" or Guam or Guyana or Haiti or Honduras or Hungary or India or Indonesia or Iran or Iraq or Jamaica or Jordan or Kazakhstan or Kenya or Korea or Kosovo or Kyrgyzstan or Laos or Latvia or Lebanon or Lesotho or Liberia or Libya or Lithuania or Macedonia or Madagascar or Malaysia or Malawi or Mali or Malta or Mauritania or Mauritius or Mexico or Micronesia or "Middle East" or Moldova or Mongolia or Montenegro or Morocco or Mozambique or Myanmar or Namibia or Nepal or "Netherlands Antilles" or "New Caledonia" or Nicaragua or Niger or Nigeria or Oman or Pakistan or Palau or Panama or "Papua New Guinea" or Paraguay or Peru or Philippines or Poland or Portugal or "Puerto Rico" or Romania or Russia or Rwanda or "Saint Kitts and Nevis" or "Saint Lucia" or "Saint Vincent and the Grenadines" or Samoa or "Saudi Arabia" or Senegal or Serbia or Montenegro or Seychelles or "Sierra Leone" or Slovenia or "Sri Lanka" or Somalia or "South Africa" or Sudan or Suriname or Swaziland or Syria or Tajikistan or Tanzania or Thailand or Togo or Tonga or "Trinidad and Tobago" or Tunisia or Turkey or Turkmenistan or Uganda or Ukraine or Uruguay or USSR or Uzbekistan or Vanuatu or Venezuela or Vietnam or Yemen or Yugoslavia or Zambia or Zimbabwe).mp. 3745213  20 18 and 19  Hits = 2183 |
| **APA PsycInfo** | 1 exp Epidemiology/ or prevalence*.mp. 276254  2 Incidence.mp. 71305  3 proportion*.ti,ab. 108717  4 rate*.ti,ab. 503768  5 occurrence.ti,ab. 56131  6 Frequency*.ti,ab. 201391  7 1 or 2 or 3 or 4 or 5 or 6 1022658  8 exp Mental Disorders/ or mental illness.mp. 1166009  9 exp Poverty Areas/ or exp Poverty/ or exp Developing Countries/ or slum*.mp. 23196  10 exp Urban Environments/ or informal settlement*.mp. 33830  11 Inner city*.mp. 6860  12 favela.mp. 102  13 exp Marginalization/ or marginalisation.mp. 6029  14 shanty town.mp. 27  15 squatter settlement*.mp. 48  16 9 or 10 or 11 or 12 or 13 or 14 or 15 65525  17 7 and 8 and 16 3319  18 limit 17 to ("200 adolescence (age 13 to 17 yrs)" or "320 young adulthood (age 18 to 29 yrs)") 1375  19 ("Low and middle income countries" or "Low income countries" or "developing countries" or BRICS or BRIC or Africa or "north africa" or "northern Africa" or "Africa South of the Sahara" or "Central Africa" or "east Africa" or "Eastern Africa" or "Southern Africa" or "Western Asia" or "central Asia" or "southeastern Asia" or "Caribbean" or "West Indies" or "South America" or "Latin America" or "Central America" or Afghanistan or Albania or Algeria or "American Samoa" or Angola or "Antigua and Barbuda" or Argentina or Armenia or Azerbaijan or Bahrain or Bangladesh or Barbados or Benin or Belarus or Belize or Bhutan or Bolivia or Bosnia-Herzegovina or Botswana or Brazil or Bulgaria or "Burkina Faso" or Burundi or Cambodia or Cameroon or "Cape Verde" or "Central African Republic" or Chad or Chile or China or Colombia or Comoros or Congo or "Costa Rica" or "Cote d'Ivoire" or Croatia or Cuba or Cyprus or Czechoslovakia or "Czech Republic" or Slovakia or Djibouti or "Democratic Republic of the Congo" or Dominica or "Dominican Republic" or "East Timor" or Ecuador or Egypt or "El Salvador" or Eritrea or Estonia or Ethiopia or Fiji or Gabon or Gambia or "Georgia" or Ghana or Grenada or Guatemala or Guinea or "Guinea-Bissau" or Guam or Guyana or Haiti or Honduras or Hungary or India or Indonesia or Iran or Iraq or Jamaica or Jordan or Kazakhstan or Kenya or Korea or Kosovo or Kyrgyzstan or Laos or Latvia or Lebanon or Lesotho or Liberia or Libya or Lithuania or Macedonia or Madagascar or Malaysia or Malawi or Mali or Malta or Mauritania or Mauritius or Mexico or Micronesia or "Middle East" or Moldova or Mongolia or Montenegro or Morocco or Mozambique or Myanmar or Namibia or Nepal or "Netherlands Antilles" or "New Caledonia" or Nicaragua or Niger or Nigeria or Oman or Pakistan or Palau or Panama or "Papua New Guinea" or Paraguay or Peru or Philippines or Poland or Portugal or "Puerto Rico" or Romania or Russia or Rwanda or "Saint Kitts and Nevis" or "Saint Lucia" or "Saint Vincent and the Grenadines" or Samoa or "Saudi Arabia" or Senegal or Serbia or Montenegro or Seychelles or "Sierra Leone" or Slovenia or "Sri Lanka" or Somalia or "South Africa" or Sudan or Suriname or Swaziland or Syria or Tajikistan or Tanzania or Thailand or Togo or Tonga or "Trinidad and Tobago" or Tunisia or Turkey or Turkmenistan or Uganda or Ukraine or Uruguay or USSR or Uzbekistan or Vanuatu or Venezuela or Vietnam or Yemen or Yugoslavia or Zambia or Zimbabwe).mp. 382528  20 18 and 19  Hits = 518 |
| **CINHAIL** | (prevalence* OR incidence* OR epidemiolog* OR frequency OR occurrence* OR rate* OR proportion) AND ("mental disorder*" OR "mental illness*" OR "psychiatric disorder*" OR "psychiatric illness*" OR "behavioural disorder*" OR "behavioral disorder*") AND (adolescen* OR youth OR teen* OR "young adult*" OR "young people" OR "young person")  AND (slum* OR "urban slum*" OR "informal settlement*" OR "urban poor" OR "urban poverty" OR "low-income communit*" OR "inner city" OR "deprived urban" OR favela* OR "shanty town*") AND ("low and middle income" OR "low-income countr*" OR LMIC OR "developing countr*" OR "Sub-Saharan Africa" OR "North Africa" OR "Central Africa" OR "East Africa" OR "Southern Africa" OR "West Africa" OR "South Asia" OR "Southeast Asia" OR "Latin America" OR "Central America" OR Caribbean OR (Afghanistan or Albania or Algeria or "American Samoa" or Angola or "Antigua and Barbuda" or Argentina or Armenia or Azerbaijan or Bahrain or Bangladesh or Barbados or Benin or Belarus or Belize or Bhutan or Bolivia or Bosnia-Herzegovina or Botswana or Brazil or Bulgaria or "Burkina Faso" or Burundi or Cambodia or Cameroon or "Cape Verde" or "Central African Republic" or Chad or Chile or China or Colombia or Comoros or Congo or "Costa Rica" or "Cote d'Ivoire" or Croatia or Cuba or Cyprus or Czechoslovakia or "Czech Republic" or Slovakia or Djibouti or "Democratic Republic of the Congo" or Dominica or "Dominican Republic" or "East Timor" or Ecuador or Egypt or "El Salvador" or Eritrea or Estonia or Ethiopia or Fiji or Gabon or Gambia or "Georgia" or Ghana or Grenada or Guatemala or Guinea or "Guinea-Bissau" or Guam or Guyana or Haiti or Honduras or Hungary or India or Indonesia or Iran or Iraq or Jamaica or Jordan or Kazakhstan or Kenya or Korea or Kosovo or Kyrgyzstan or Laos or Latvia or Lebanon or Lesotho or Liberia or Libya or Lithuania or Macedonia or Madagascar or Malaysia or Malawi or Mali or Malta or Mauritania or Mauritius or Mexico or Micronesia or "Middle East" or Moldova or Mongolia or Montenegro or Morocco or Mozambique or Myanmar or Namibia or Nepal or "Netherlands Antilles" or "New Caledonia" or Nicaragua or Niger or Nigeria or Oman or Pakistan or Palau or Panama or "Papua New Guinea" or Paraguay or Peru or Philippines or Poland or Portugal or "Puerto Rico" or Romania or Russia or Rwanda or "Saint Kitts and Nevis" or "Saint Lucia" or "Saint Vincent and the Grenadines" or Samoa or "Saudi Arabia" or Senegal or Serbia or Montenegro or Seychelles or "Sierra Leone" or Slovenia or "Sri Lanka" or Somalia or "South Africa" or Sudan or Suriname or Swaziland or Syria or Tajikistan or Tanzania or Thailand or Togo or Tonga or "Trinidad and Tobago" or Tunisia or Turkey or Turkmenistan or Uganda or Ukraine or Uruguay or USSR or Uzbekistan or Vanuatu or Venezuela or Vietnam or Yemen or Yugoslavia or Zambia or Zimbabwe)  Hits =144 |
| **SCOPUS** | (ALL (prevalenc* OR incidenc* OR epidemiolog* OR ratio* OR proportio* OR rate* OR frequenc* ) AND ALL (Mental Disorders OR Behavioural and mental disorders OR Mental illness) AND ALL  (poverty OR low-income OR low socioeconomic OR disadvantaged OR slum OR informal settlement OR urban poor OR inner city OR deprived urban OR Favela OR shanty town OR squatter settlement) AND ALL (adolescen* OR youth OR "young people" OR "young person" OR teenager* ) AND ALL ("Low and middle income countries" OR "Low income countries" OR "developing countries" OR BRICS OR BRIC OR Africa OR "north africa" OR "northern Africa" OR "Africa South of the Sahara" OR "Central Africa" OR "east Africa" OR "Eastern Africa" OR "Southern Africa" OR "Western Asia" OR "central Asia" OR "southeastern Asia" OR "Caribbean" OR "West Indies" OR "South America" OR "Latin America" OR "Central America")  Hits =35 |
| **Web of Science** | TS=(prevalence* OR incidence* OR epidemiolog* OR proportion OR rate* OR frequency OR occurrence*) AND TS=("mental disorder*" OR "mental illness*" OR "mental health problem*" OR "behavioural disorder*" OR "behavioral disorder*" OR "psychiatric disorder*" OR "emotional disorder*") AND TS=(youth OR adolescen* OR teen* OR "young adult*" OR "young people" OR "young person") AND TS=(slum* OR "urban slum*" OR "informal settlement*" OR "urban poor" OR "poor urban" OR "urban poverty" OR "low-income communit*" OR "deprived urban" OR favela* OR "shanty town*") AND TS=("low- and middle-income" OR "low and middle income" OR LMIC OR "developing countr*" OR "low-income countr*" OR "middle-income countr*" OR "Sub-Saharan Africa" OR "North Africa" OR "Central Africa" OR "East Africa" OR "Eastern Africa" OR "Southern Africa" OR "West Africa" OR "Western Africa" OR "South Asia" OR "Southeast Asia" OR "Central Asia" OR "Western Asia" OR "Middle East" OR "Latin America" OR "South America" OR "Central America")  Hits = 256 |
| **African Journals ONLINE** | Mental health AND slum OR informal settlement OR urban poor  Hits = 84 |

**SUPPLEMENT 2**

Risk of bias assessment for individual studies using JBI

| **S/N** | **Author and year** | **Sample frame** | **Appropriate sampling** | **Sample size** | **Description of subjects/setting** | **Data Analysis** | **Valid methods for identification of condition** | **Standard and reliable measure of condition for all participants** | **Statistical analysis** | **Adequate response rate** | **Total score** | **Risk of Bias** |
| --- | --- | --- | --- | --- | --- | --- | --- | --- | --- | --- | --- | --- |
| 1 | Avanci et al 2021 | Y | Y | Y | Y | Y | Y | Y | Y | U | 8 | Low |
| 2 | Cheng et al 2014(a) | N | U | Y | U | Y | U | Y | Y | U | 4 | High |
| 3 | Cheng et al 2014(b) | N | U | Y | U | Y | U | Y | Y | U | 4 | High |
| 4 | Cheng et al 2014(c) | N | U | Y | U | Y | U | Y | Y | U | 4 | High |
| 5 | Cheng et al 2014 (d^)^ | N | U | Y | U | Y | U | Y | Y | U | 4 | High |
| 6 | Gibbs st al 2018 | N | N | U | Y | Y | Y | Y | N | U | 4 | High |
| 7 | Harder et al 2012 | Y | Y | Y | Y | Y | Y | Y | Y | U | 8 | Low |
| 8 | Nasreen et al 2018 | Y | Y | Y | Y | Y | Y | Y | Y | Y | 9 | Low |
| 9 | Ndungu et al 2020 | N | N | Y | Y | Y | Y | Y | Y | U | 6 | Low |
| 10 | Bantjes et al, 2018 | Y | Y | Y | Y | Y | Y | Y | Y | Y | 9 | Low |
| 11 | Barman et al 2015 | Y | Y | Y | U | Y | Y | U | N | U | 5 | Moderate |
| 12 | Hatcher et al 2019 | N | N | Y | Y | Y | Y | Y | Y | U | 6 | Low |
| 13 | Mridha et al 2021 | Y | Y | Y | U | Y | Y | Y | Y | Y | 8 | Low |
| 14 | Rani et al, 2018 | Y | Y | Y | Y | Y | Y | Y | Y | Y | 9 | Low |
| 15 | Somrongthong et al 2013 | Y | Y | Y | Y | Y | Y | Y | Y | U | 7 | Low |
| 16 | Wado et al 2022 | Y | Y | Y | Y | Y | Y | U | Y | Y | 8 | Low |
| 17 | Daniel et al 2022 | Y | Y | Y | Y | Y | Y | Y | Y | Y | 9 | Low |
| 18 | Friedberg et al 2023 | Y | Y | Y | Y | Y | Y | Y | Y | U | 8 | Low |
| 19 | Kaufman et al 2014 | Y | Y | Y | Y | Y | Y | Y | Y | Y | 9 | Low |
| 20 | Swahn et al 2025 | U | N | U | Y | Y | Y | Y | Y | U | 5 | Moderate |
| 21 | Mahanta et al 2026 | Y | N | Y | Y | Y | Y | Y | U | U | 6 | Low |
| 22 | Musyoka et al 2025 | U | N | U | Y | U | Y | Y | U | Y | 4 | High |
| 23 | Oyekunle et al 2023 | Y | N | Y | Y | Y | Y | Y | Y | Y | 8 | Low |
| 24 | Vuyk et al 2025 | U | U | U | Y | Y | Y | Y | Y | U | 5 | Moderate |

**SUPPLEMENT 3**

Funnel plot for Depression studies
